# A Method to Visualize and Quantify the Intraosseous Arteries of the Femoral Head by Vascular Corrosion Casting

**DOI:** 10.1111/os.13319

**Published:** 2022-07-11

**Authors:** XiangNan Zhang, Wei Deng, JiHui Ju, Songqiang Zhang, HongYu Wang, KaiLong Geng, DingSong Wang, GuangLiang Zhang, YingYing Le, RuiXing Hou

**Affiliations:** ^1^ Department of Orthopaedics Suzhou Ruihua Orthopedic Hospital Suzhou China; ^2^ Suzhou Medical College of Soochow University Suzhou China; ^3^ Teaching Hospital of Medical College of Yangzhou University, Suzhou Ruihua Orthopedic Hospital Suzhou China; ^4^ CAS Key Laboratory of Nutrition, Metabolism and Food Safety Shanghai Institute of Nutrition and Health, University of Chinese Academy of Sciences, Chinese Academy of Sciences Shanghai China

**Keywords:** Arteries, Corrosion casting, Femoral head, Intraosseous, Perfusion, Swine

## Abstract

**Objective:**

To describe a method to display the three‐dimensional distribution of intraosseous arteries in the femoral head by vascular corrosion casting.

**Methods:**

An experimental study was done to expose the intraosseous arteries of the femoral head by a microperfusion corrosion method between January 2021 and May 2021. Specimens were 23 swine femoral heads (12 female specimens and 11 male specimens, where age of swine ranged from 8 to 12 months, and the weight was approximately 150 kg). The femoral heads were microperfused with the vascular casting resin through retinacular arteries, and the bone of the femoral head was dissolved with 50% sodium hydroxide and 10% hydrochloric acid and rinsed under the microscope until the vessel casts were completely exposed. The distribution and anastomosis of the arteries in the femoral head were observed under direct vision and microscopy. The diameter of the artery in the femoral head was measured at 0.5 cm after its entry into the bone of the femoral head with a microscale under the microscope. The number of internal arteries with diameter ≥0.05 mm was counted. The number and diameter of the main trunk of the epiphyseal arteries in the femoral head between male and female swine were compared.

**Results:**

The vascular casting specimen of the swine femoral head was successfully produced by using epoxy resin as a casting agent, and the three‐dimensional intraosseous vascular structures were clearly visible. The number of epiphyseal arteries in male and female swine was 8.55 ± 2.15 and 8.83 ± 2.15 (*t* = −0.31, *p* = 0.38), respectively. The diameters of the superior epiphyseal arteries in male and female swine were 0.35 ± 0.09 and 0.31 ± 0.08 mm (*t* = 1.03, *p* = 0.16), the diameters of the inferior epiphyseal arteries were 0.47 ± 0.05 and 0.49 ± 0.09 mm (*t* = −0.57, *p* = 0.29), and the diameters of the anterior epiphyseal arteries were 0.34 ± 0.08 and 0.33 ± 0.13 mm (*t* = 0.32, *p* = 0.37). There was no significant difference in the number and diameter of the main trunk of intraosseous arteries between male and female swine (*p* > 0.05). The main trunk of intraosseous arteries formed an anastomosis in the center of the femoral head. Among 23 swine femoral head samples, three types of intraosseous anastomosis were observed, including 13 (57%) posterior superior‐posterior inferior, seven (30%) posterior inferior‐anterior, and three (13%) uniform intraosseous anastomosis.

**Conclusion:**

The microperfusion corrosion method can produce the vascular casting specimen of swine femoral head revealing the three‐dimensional structure of the intraosseous artery, which clearly shows the origin, course and branches, and diameter, as well as the anastomosis, of nutrient arteries in the femoral head. This method provides a simple and rapid technique for quantifying and visualizing intraosseous arteries.

## Graphical abstract

To visualize and quantify the intraosseous arteries of the femoral head. We described a method to visualize the three‐dimensional distribution of the intraosseous arteries by vascular corrosion casting.

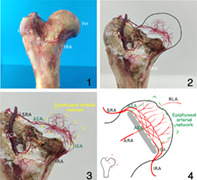

## Introduction

Most of the femoral neck fractures in young adults are caused by high‐energy impact, and the common types of the fracture are Garden III and IV. The vascular damage resulting from fracture displacement is the main cause of avascular necrosis of the femoral head or nonunion of femoral neck after femoral neck fracture. At present, the distribution of blood vessels outside the femoral head is well‐known. Anatomical studies have shown that the retinacular arteries are a main source of blood supply to the femoral head,^1^, ^2^, ^3^ but the distribution of blood vessels in the femoral head is not fully known.^4^, ^5^, ^6^ Osteonecrosis of the femoral head after femoral neck fracture and femoral neck fracture healing are thought to be related to vascular anatomy. Understanding the precise distribution and characteristics of intraosseous arteries of the femoral head will be helpful for developing novel therapeutic strategies against osteonecrosis in patients with femoral neck fracture.

Studies of intraosseous arteries of the femoral head have traditionally been challenging because of the hard, calcified structure of bone. The traditional method used to display blood vessels in the femoral head is the Spalteholz transparent technique. It is complex and time‐consuming and cannot clearly show the three‐dimensional structure of the internal arteries of the femoral head.^7^ Recently, angiographic methods and micro‐CT scans have been used to reconstruct the three‐dimensional structures of the intraosseous blood supply in human femoral heads.^8^, ^9^, ^10^ This method shows blood vessels in the femoral heads in an indirect way, requires expensive equipment, and may result in CT scan artifacts. Therefore, neither the traditional vascular exposure method nor the use of videography techniques can better display the intraosseous arteries of the femoral head. There is an urgent need for a method that can clearly, directly, and three‐dimensionally visualize the intraosseous arteries of the femoral head and that could accurately and quantitatively analyze the distribution of the intraosseous arteries.

According to reports, the swine femoral head has many similarities in appearance with the human femoral head, including the greater trochanter, lesser trochanter, femoral neck, femoral head, and round ligament. In addition, in terms of blood vessels, both of them include the superior, inferior, and anterior retinacular arteries, the round ligament arteries, and the intraosseous nourishing arteries. Therefore, the swine femoral head is a good basic research model.^11^

The present study aimed to investigate (i) whether the strategy could establish a method to expose the intraosseous arteries of the femoral head; (ii) whether the casting specimen of intraosseous arteries could be made by using epoxy resin as a casting agent; and (iii) whether the anatomical characteristics of intraosseous arteries could be studied by using casting specimens of intraosseous arteries. It is hypothesized that the strategy could establish a method to expose blood vessels in the swine femoral head by microperfusion with a vascular casting medium and corrosion of the bone with sodium hydroxide and hydrochloric acid. The present study found that the three‐dimensional distribution of the internal arteries of the femoral head was clearly visualized and the number and diameter of the main arteries were precisely quantified.

## Materials and Methods

### Materials

Twenty‐three specimens consisting of hip joint with capsule and blood vessels and the proximal part of the femur were collected from 12 swine that were sacrificed. Twelve specimens were from six females and 11 specimens were from six males. The age of swine was from 8 to 12 months (mean 10 months), and the body weight was approximately 150 kg. This study was approved by the Ethics Committee of Ruihua Hospital affiliated with Soochow University (approval number RX2019003).

Epoxy resins were purchased from the Dongguan Xiaoma New Material Technology Co., Ltd. (Dongguan, China) (Cat No. 318AB‐C2‐1 kg). The vascular casting medium was prepared by mixing epoxy A solution and epoxy B solution (3:1, V/V) followed by adding eight drops of red metal complex dye. The vascular casting medium was used to perfuse arteries between 30 and 60 min after preparation.

### Vascular Casting

Capsulotomy was performed along the rim of acetabulum, then the ligament of the femoral head was cut, and the acetabulum was removed. The main trunks of the superior, inferior, and anterior retinacular arteries were visualized microscopically and perfused with normal saline containing 625 U/ml heparin to flush the blood in the femoral heads. The broken branches of the arteries were ligated. About 3 ml of vascular casting medium was injected under appropriate pressure into each trunk of the retinacular arteries. An appropriate amount of casting medium was injected every 15 min to keep the retinacular artery full. The perfused femoral head was kept at room temperature for 24 h. The tissues surrounding the blood vessels were then excised.

### Corrosion

**Fig. 1:**
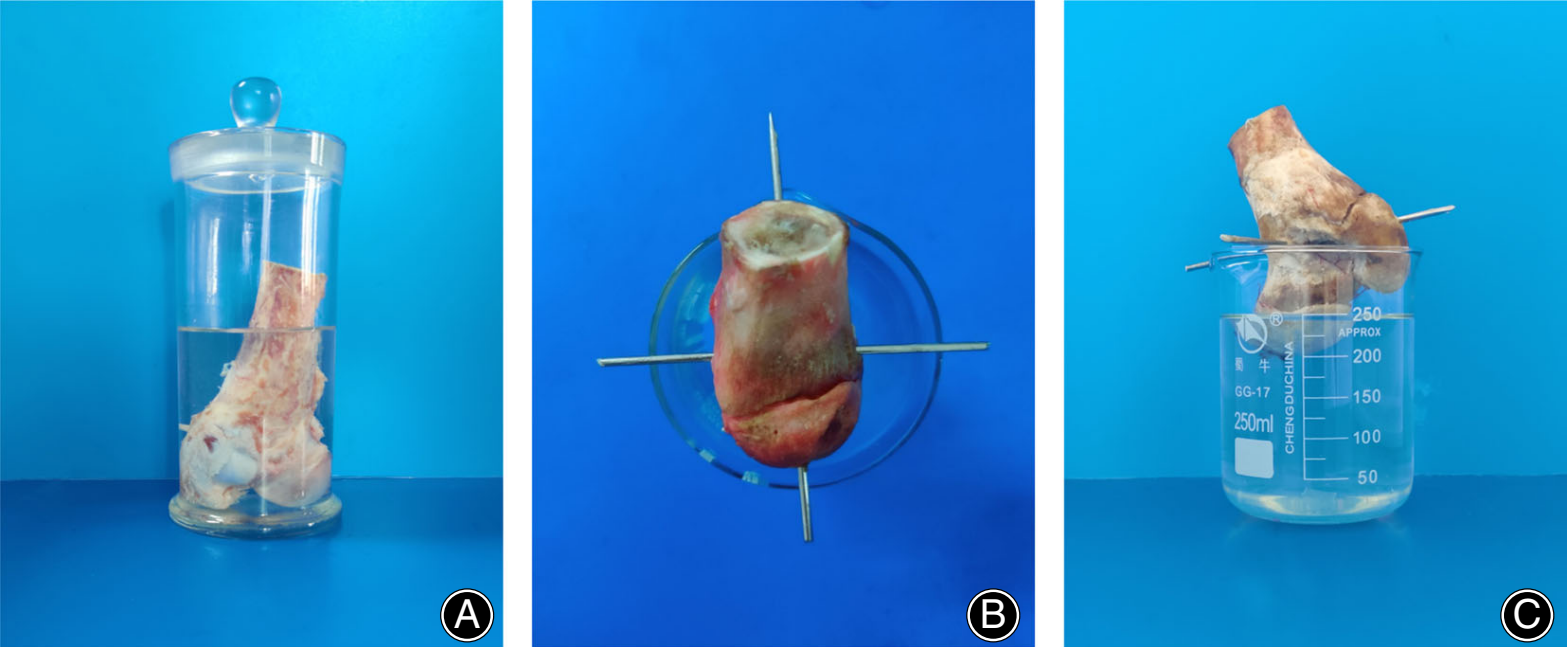
Corrosion of the bone of swine femoral head by alkali and acid. Photographs of the femoral head immersed in 50% sodium hydroxide (A), fixed with Kirschner pins (B), and immersed in 10% hydrochloric acid (C)

**Fig. 2:**
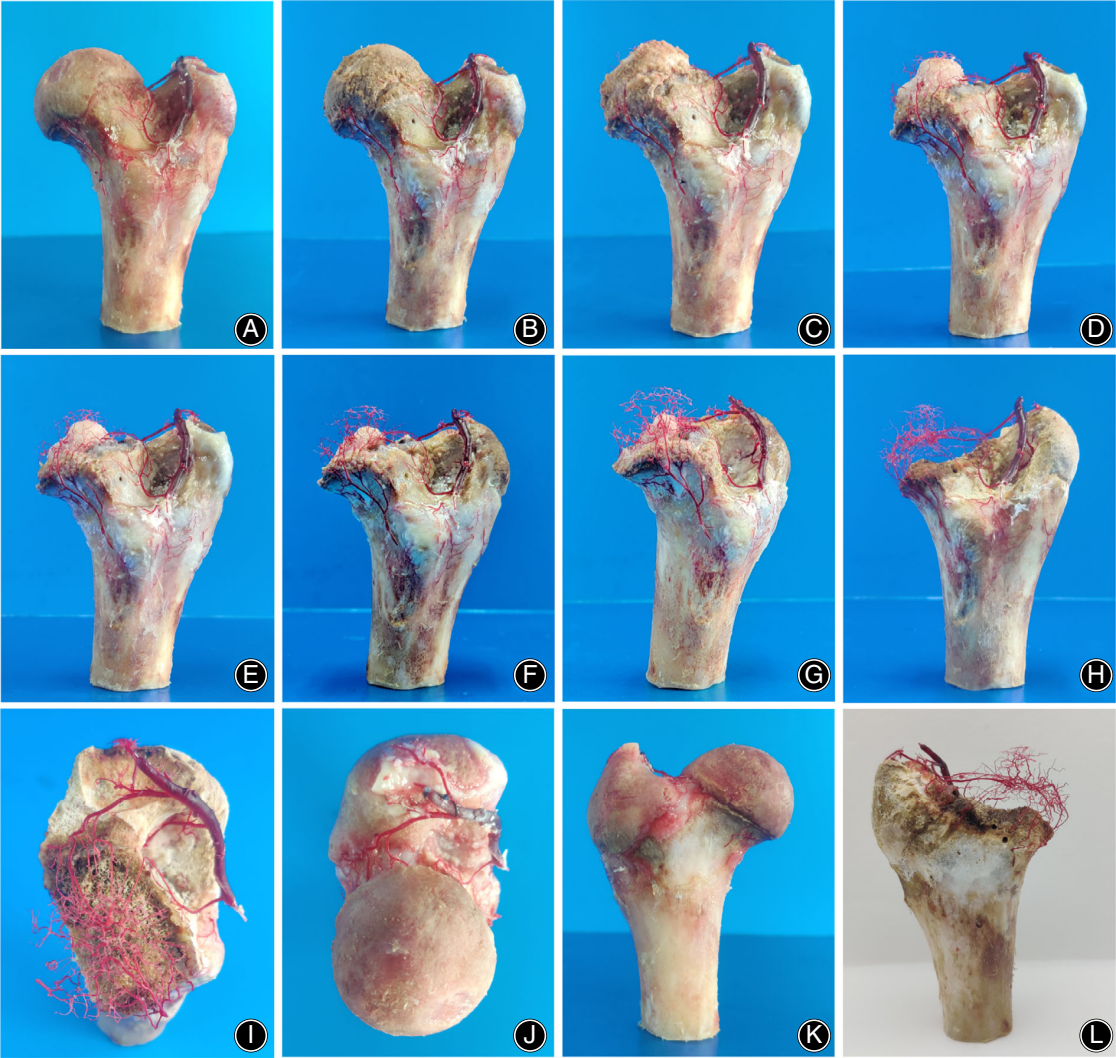
Exposure of blood vessels in swine femoral head in different stages of chemical corrosion of the bone. (A‐B) Photographs of vascular structures after incubation of the femoral head with 50% sodium hydroxide (A) and 10% hydrochloric acid (B). (C‐H) Photographs of vascular structures after corrosion of the bone with 10% hydrochloric acid for 1–6 times and rinsing with water. (I‐L) Comparison of internal bone arteries before and after exposure

**Fig. 3:**
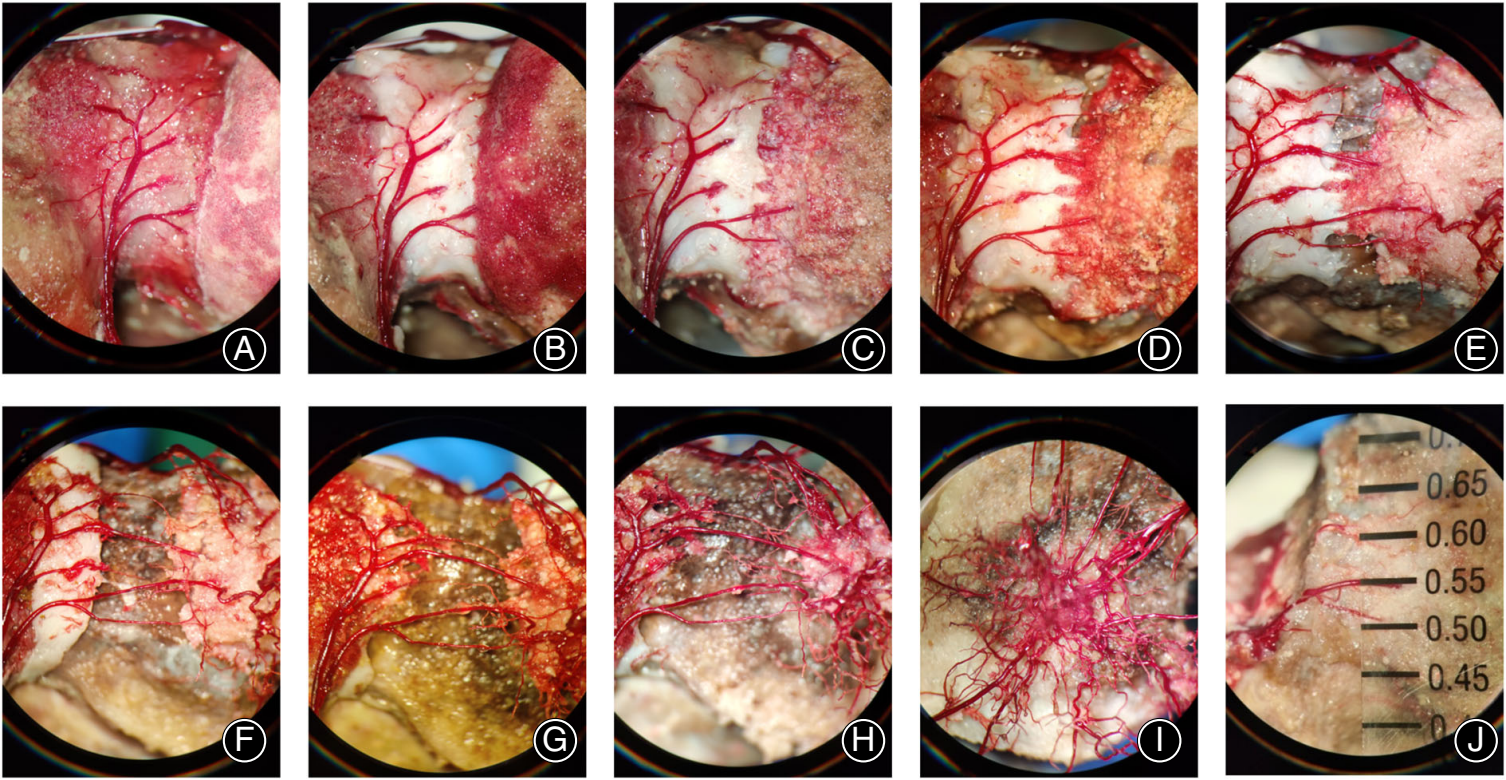
Exposure of blood vessels in swine femoral head in different stages of chemical corrosion of the bone. (A‐B) Photographs of vascular structures after incubation of the femoral head with 50% sodium hydroxide (A) and 10% hydrochloric acid (B). (C‐H) Photographs of vascular structures after corrosion of the bone with 10% hydrochloric acid for 1–6 times and rinsing with water. (I) Corrosion and washing are completed, and impurities are removed. (J) Photograph showing an artery with caliber of 0.55 mm in the femoral head. Picture I was taken at an angle perpendicular to the axis of the femoral neck. The magnification of all photos is 10

The femoral head was macerated upside down in 50% sodium hydroxide (NaOH) solution, about 5 cm from the lesser trochanter (Figure 1A). After 6 h, the tissues surrounding the vessel cast were separated and excised under the microscope. The femoral head was rinsed with tap water. The femur was truncated at 3 cm beneath the inferior edge of the lesser trochanter. The femoral head was fixed with two crossed 1.5‐mm Kirschner pins at the interface of femoral neck and head (Figure 1B). The femoral head was immersed in 10% HCl solution (Figure 1C) for 12–24 h and rinsed with tap water under the microscope by turns until the vessel casts were completely exposed (Figures 2 and 3). The 10% HCl solution was replaced each time.

### Measurement of Internal Arteries of Femoral Head

The distribution and anastomosis of the arteries in the femoral head were observed under the microscope. The diameter of the artery in the femoral head was measured at 0.5 cm after its entry into the bone of femoral head with a microscale under the microscope (Figure 3J). The number of internal arteries with diameter ≥0.05 mm was counted.

### Statistical Analysis

The data were expressed as mean ± standard deviation (SD). Statistical analysis was performed with Excel software. An independent two‐sample *t*‐test was performed to compare the number and diameter of the main trunk of epiphyseal arteries in the femoral head between male and female swine. The value of *p* < 0.05 was considered as statistically significant.

## Results

### Characteristics of Vascular Casting Medium

The casting medium composed of epoxy resins was an atoxic liquid that could be easily injected into blood vessels with an inner diameter of 0.05 mm. After testing several times, it was found that the best time to perfuse the artery with casting medium was 30–60 min after its preparation. After 1.5 h of preparation, the casting medium became sticky and was difficult to inject through a cannula. After 24–36 h of preparation, the casting medium became a solid with high strength and good supporting capacity.

### Three‐Dimensional Distribution and Diameter of Internal Arteries of Femoral Head

**Fig. 4:**
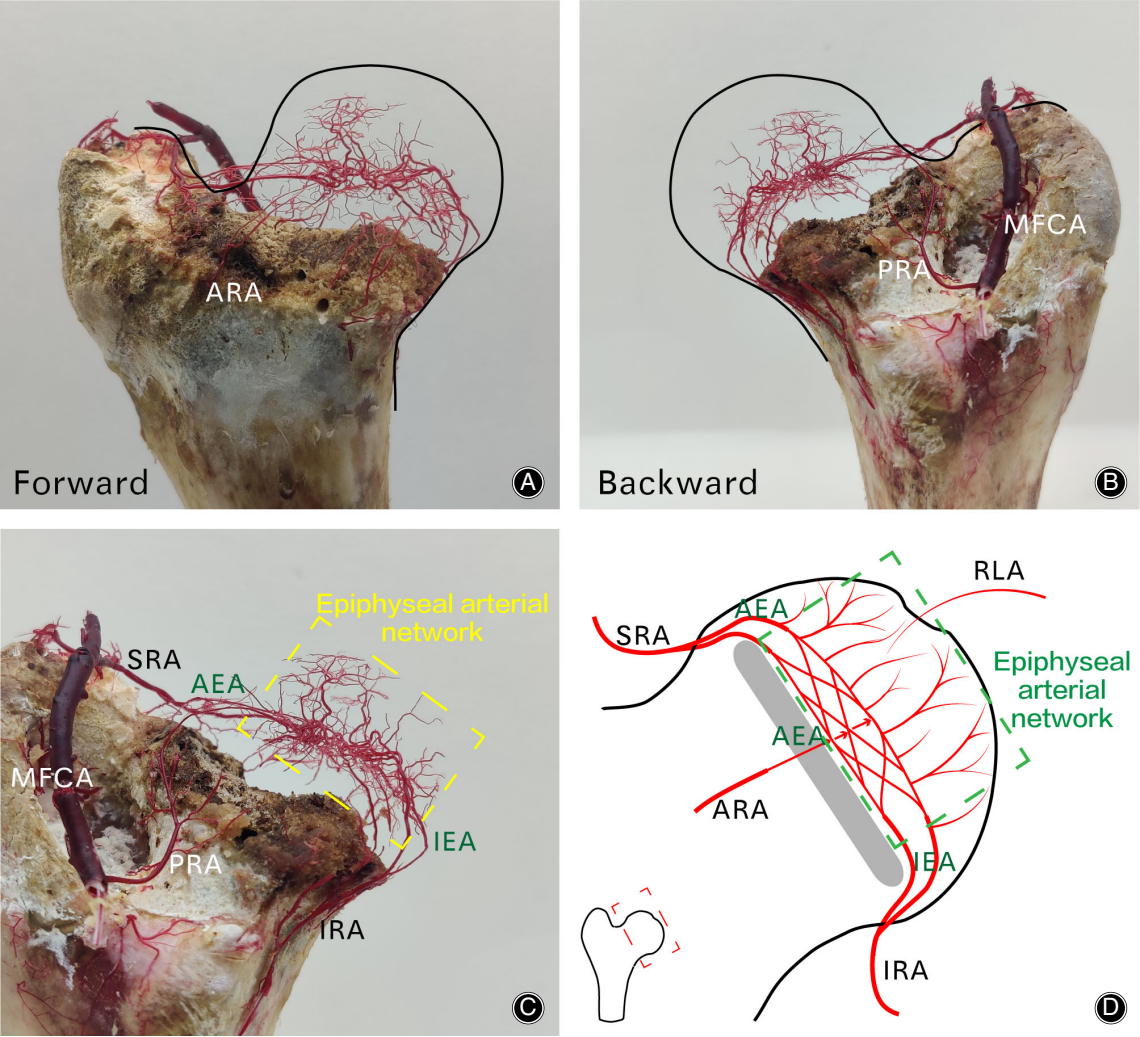
Physical image and schematic diagram of internal femoral head artery. (A‐B) Physical image of the internal artery of the femoral head; (C‐D) Comparison of physical image and schematic diagram of internal femoral head artery. RLA, round ligament artery; SRA, superior retinacular artery; IRA, inferior retinacular artery; ARA, anterior retinacular artery; PRA, Posterior retinacular artery; SEA, superior epiphyseal artery; IEA, inferior epiphyseal artery; AEA, anterior epiphyseal artery; MFCA, the medial femoral circumflex artery

**Fig. 5:**
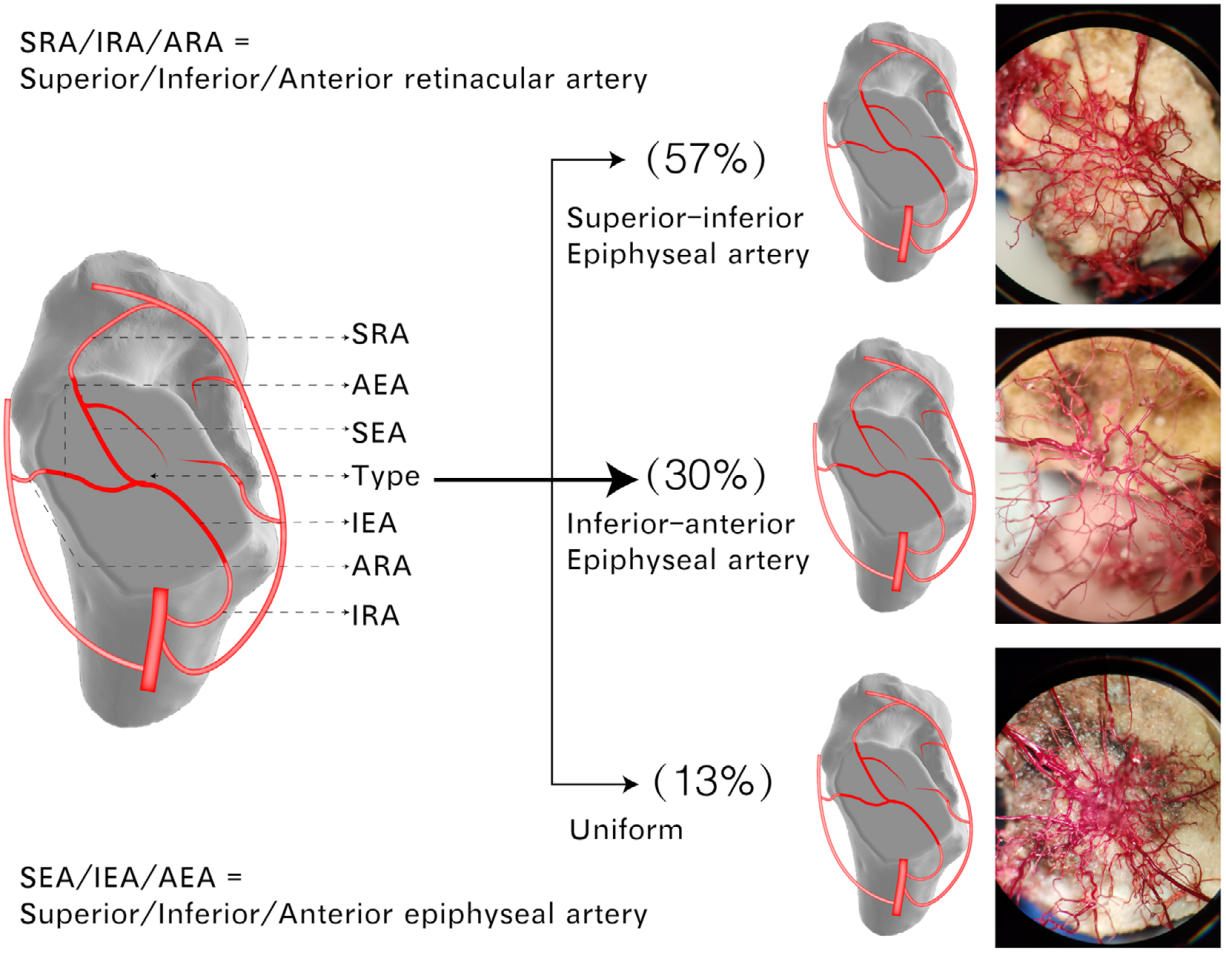
Gross view of the three‐dimensional distribution of arteries in the femoral head. Photographs showing three types of anastomoses of the arteries in the femoral head: superior–inferior, inferior‐anterior, and uniform anastomoses. The proportion of three types of epiphyseal artery anastomosis in 23 femoral heads

After corrosion with NaOH and HCl, the retinacular arteries and branches in the femoral head were gradually exposed (Figures 2 and 3). Figure 4 and Video1 show general (Figure URE 4A–D) and microscopic (Figure 5) views of the three‐dimensional distribution of the internal arteries in the femoral head. The entrance of the retinacular arteries into the bone, the branches of retinacular arteries, and the vascular anastomosis in the femoral head are clearly visible. The superior, inferior, and anterior retinacular arteries entered the femoral head at the interface of the femoral neck and head, where each had a main trunk and one or two small branches. The number of epiphyseal arteries in male and female swine was 8.55 ± 2.15 and 8.83 ± 2.15 (*t* = −0.31, *p* = 0.38), respectively. The diameters of the superior epiphyseal arteries in male and female swine were 0.35 ± 0.09 and 0.31 ± 0.08 mm (*t* = 1.03, *p* = 0.16), the diameters of the inferior epiphyseal arteries were 0.47 ± 0.05 and 0.49 ± 0.09 mm (*t* = 0.57, *p* = 0.29), and the diameters of the anterior epiphyseal arteries were 0.34 ± 0.08 and 0.33 ± 0.13 mm (*t* = 0.32, *p* = 0.37). There was no significant difference in the number and diameter of the main trunk of intraosseous arteries between male and female swine (*p* > 0.05). The dates are listed in Table 1. After entering the femoral head, the main trunk of the retinacular artery ran along the periphery in the femoral head and went to the center of the femoral head in an almost vertical direction after crossing the epiphyseal plate. On its course, the artery gave off several branches. Upon reaching the center of the femoral head, the main branches formed an anastomosis. Among 23 swine femoral head samples, three types of intraosseous anastomosis were observed, including 13 (57%) posterior superior‐posterior inferior, seven (30%) posterior inferior‐anterior, and three (13%) uniform intraosseous anastomosis (Figure 5).

**TABLE 1:** The number and diameter of main trunk epiphyseal arteries in the femoral head (mean ± SD)

Sex	Number	Diameter (mm)			
	Main trunk epiphyseal arteries	Superior epiphyseal artery	Inferior epiphyseal artery	Anterior epiphyseal artery	
Male (*n* = 11)	8.55 ± 2.15	0.35 ± 0.09	0.47 ± 0.05	0.34 ± 0.08
Female (*n* = 12)	8.83 ± 2.15	0.31 ± 0.08	0.49 ± 0.09	0.33 ± 0.13
*t*	−0.31	1.03	−0.57	0.32
*p*	0.38	0.16	0.29	0.37

## Discussion

### Clinical Significance of the Microperfusion Corrosion Method

It is important to visualize and quantify the intraosseous arteries of the femoral head because the blood supply is essential to every cell.^8^ Various diseases have been reported to affect the blood supplies to intraosseous arteries, for example, osteonecrosis of the femoral head, lunate osteonecrosis, humeral head necrosis, and navicular osteonecrosis, which indicates that the anatomy of intraosseous arteries urgently needs to be clarified. However, the study of intraosseous vasculature has always been challenging because of the hard and calcified structure of bone.

In this study, the swine femoral head was used as a model to develop a method for vascular casting and exposure of the internal arteries of the femoral head. The retinacular arteries of the hip joint were separated and intubated under the microscope and infused with the epoxy resin. The intraosseous arteries were exposed by corrosion of the bone with acid and alkali. The origin, three‐dimensional distribution and anastomosis of the internal arteries of the femoral head were clearly visible. Detailed studies of intraosseous arteries will be useful for understanding diseases due to damage to blood supply and will help doctors to preserve the blood supplies of the femoral head during surgery and therefore, reduce iatrogenic osteonecrosis of the femoral head. The use of this method can aid the understanding of the mechanisms underlying bone necrosis, osteoporosis, fracture healing, and so forth.

### Importance of Knowledge about the Intraosseous Vasculature

It is very important to clarify the blood supply of the femoral head because the blood supply plays an important role in the occurrence and development of femoral head necrosis.^12^ The use of the microperfusion corrosion method can better show the blood supply of the femoral head and help surgeons protect the blood supply of the femoral head during surgery, thereby reducing the occurrence of iatrogenic femoral head necrosis and nonunion.^8^ For example, when treating femoral neck fractures, implanted internal fixation is frequently used, and sometimes the implantation of internal fixation may damage the blood supply of the femoral head. In this study, the diameter of the inferior epiphyseal artery was found to be 0.47 ± 0.08 mm (male and female), which is the thickest branch of the three epiphyseal arteries. The epiphyseal artery appears in a specific area (a subject for future study), so the screw used in femoral neck fracture internal fixation that enters the femoral head should not damage the inferior epiphyseal artery. Implantation of internal fixation avoiding the main epiphyseal arteries of the femoral head may reduce iatrogenic femoral head necrosis, which will be further studied in the future.

In femoral neck fractures, the superior retinacular arteries are more likely to be damaged, while the inferior retinacular arteries have a greater chance of remaining intact. At this time, the blood supply of the femoral head mainly comes from the inferior retinacular arteries, so it is necessary to protect the inferior retinacular arteries and inferior epiphyseal arteries during the procedure. Therefore, clarifying the characteristics of the blood supply distribution in the femoral head is helpful to study related diseases caused by the blood supply damage in the femoral head. In the current study, the blood vessels with a diameter of 0.05 mm could be observed and measured under the microscope. In addition, the present study showed that there were three types of vascular anastomosis in swine femoral heads, and among them the posterior superior‐posterior inferior type accounts for 57% (Figure 5). Therefore, the blood supply of the swine femoral head mainly comes from the posterior superior‐posterior inferior retinacular arteries.

The number of epiphyseal arteries in male and female swine was 8.55 ± 2.15 and 8.83 ± 2.15 (*t* = −0.31, *p* = 0.38), respectively. The diameters of the superior epiphyseal arteries in male and female swine were 0.35 ± 0.09 and 0.31 ± 0.08 mm (*t* = 1.03, *p* = 0.16,), the diameters of the inferior epiphyseal arteries were 0.47 ± 0.05 and 0.49 ± 0.09 mm (*t* = −0.57, *p* = 0.29), and the diameters of the anterior epiphyseal arteries were 0.34 ± 0.08 and 0.33 ± 0.13 mm (*t* = 0.32, *p* = 0.37). There was no significant difference in the number and diameter of the main trunks of intraosseous arteries in the femoral head between male and female swine. In a previous study, Qiu *et al*.^8^ compared the number and diameter of femoral head arteries on both sides of the human femoral head and found no statistical difference between the two. This means that when treating femoral head diseases caused by blood vessels, left or right or gender are not factors to be considered.

It has been reported that the main blood supply for the human femoral head is from the superior and inferior retinacular arteries.^4^, ^13^ Therefore, the blood supply for the femoral head is similar between swine and humans. The superior epiphyseal artery of the human femoral head is the thickest,^9^ while the thickest in the swine femoral head is the inferior epiphyseal artery. This is the difference between the two. Zhao *et al*.^14^ performed digital subtraction angiography (DSA) on a 24‐year‐old patient with femoral neck fracture and found that the superior retinacular artery was severed. During the operation, the superior retinacular artery was anastomosed and the blood supply to the femoral head was restored. If the end of the retinacular artery is located at the junction of the head and neck, it cannot be anastomosed directly. In this study, the diameter of the main epiphyseal artery was usually more than 0.3 mm. If a groove can be made at the femoral head nourishing hole to expose the epiphyseal artery in the bone, it is possible to restore the blood supply of the femoral head.^11^ This view will be studied in the future.

Osteonecrosis of the femoral head often occurs in the posterolateral area of the femoral head, the weight‐bearing area of the femoral head. This area is in dynamic balance between injury and repair. In this study, it was found that the epiphyseal artery network of the femoral head is located more toward the posterolateral area (the apex area of the femoral head, see Figure 5A–D). This further shows that a good blood supply is compatible with bone repair. When external factors (such as femoral neck fracture, hormone application, and decompression sickness) cause the blood supply of the femoral head to decrease, the balance between damage and repair based on the blood supply is upset, and therefore the posterolateral area of the femoral head is susceptible to osteonecrosis.

The present study found that the superior, inferior, and anterior epiphyseal arteries are directly anastomosed in the form of the main trunk in the femoral head, which ensures that the blood supply inside the femoral head has a high degree of connectivity. The area of the epiphyseal artery network, in addition to the main anastomosis, also contains more branches of mutual anastomosis (Figures 2I, 3I, and 4D). These communication branches also ensure the connectivity of the blood supply of the femoral head. After a femoral neck fracture occurs, some patients with displaced femoral neck fractures do not develop osteonecrosis, which may be a result of the blood supply of the femoral head being slightly compensated for by the vascular network of the femoral head (Figure 4D).

### Conventional Methods Used to Study Intraosseous Vasculature

The study of intraosseous vasculature has always been challenging because of the hard and calcified structure of bone. The traditional method used to visualize blood vessels in tissues and organs is the Spalteholz transparency technique, which makes bones and other tissues transparent. However, decalcification of the femoral head using the Spalteholz transparency technique may take several months. The modified Spalteholz transparency technique improves the visibility and integrity of intraosseous blood vessels,^3^ but is still time‐consuming and makes it difficult to quantify aspects of the blood vessels, such as vascular diameter. Papakonstantinou *et al*.^15^ injected 6% hydrogen peroxide‐diluted ink into the intraosseous arteries and tracked the course of intraosseous arteries and their branches by removing the bone with hammer, chisel, rongeur, and mounting needle. This method saves time in exposing the intraosseous arteries but may damage the arteries, and the diameter and number of intraosseous arteries cannot be quantified.

In the present study, strong acid and alkali were used to corrode bone tissue, which greatly shortened the time to 1 week for exposing the intraosseous blood vessels. Compared with the method used by Papakonstantinou *et al*.,^15^ corrosion of bone tissue with acid and alkali greatly reduced the damage to small blood vessels, and the intraosseous arteries were fully visualized. Therefore, the microperfusion corrosion method for the study of internal bone arteries can be applied to the study of multiple intraosseous blood vessels in the body such as the human femoral head, carpal bone, and talus.

### Microperfusion Corrosion Method Used in this Study

Vascular corrosion casting is an important technique to study the distribution of blood vessels in tissues and organs. The commonly used vascular casting reagents are latex, ethylene resin, vinyl perchloride, and self‐coagulating denture powder.^16^, ^17^, ^18^ Latex is often used to track the course of blood vessels after vascular perfusion, but it cannot form a cast after vascular corrosion. The vascular cast made by using ethylene resin and perchloroethylene is soft and susceptible to collapse. The casting medium made by self‐coagulating denture powder is hard to pass through the vessels with a diameter less than 0.1 mm, and the vascular cast made by using this material is fragile. The polyurethane foam casting reagent rapidly solidifies after being injected into blood vessels,^19^ and it cannot be used to replenish the poor‐filling vessels. The epoxy resin used in this study is stable and atoxic and widely used in the manufacture of handicrafts.

The vascular casting medium was prepared by mixing epoxy resin A and B solutions and injecting the mixture into blood vessels after 30–60 min when its viscosity was not too high to pass through the blood vessels. The vessels with poor filling could be perfused with more casting medium within 4 h. The previous study found that compared with vinyl perchloride, the strength of solidified epoxy resin was higher, and the structures of vascular casts with diameter of 0.1 mm were better (data not shown). The procedure in the present method to study the three‐dimensional distribution of intraosseous blood vessels in the femoral head is simple and easy to apply, with no need for special instruments. This method can also be used to study the vascular structure of other organs and tissues. Another study is using the microperfusion corrosion method established in this study to investigate human femoral head arteries and veins, arteriovenous integrated vascular casts, and intraosseous vessels of the humeral head and the distal radius.

### Limitations

The limitations of this method are as follows: (i) The vascular system within the femoral head includes both arteries and veins, and veins were not involved in this study. At present, another study has used this method to perfuse the veins and make vascular casting specimens within the femoral head including arteries and veins. (ii) The diameter of the vascular cast may be smaller than the actual vessel because of the shrinkage of epoxy resin after polymerization. From the current research, most casting agents (latex, ethylene resin, vinyl perchloride, and self‐coagulating denture powder) shrink. The data reduction caused by shrinkage can be ignored because the shrinkage of epoxy resin is small; and (iii) some microvessels may be damaged and lost during rinsing of the corroded bone. This is probably unavoidable in any casting study, where vessels 0.05 mm in diameter and below are preserved (Figure 4 and Video1). During flushing, the water flow should be fine and slow enough to retain more microvessels. In addition, this study was limited to swine femoral heads with a limited sample size, which can provide only an intraosseous vascular casting method and an animal experimental model of femoral head necrosis. In future research, the sample size should be increased and the vascular network of the human femoral head should be studied to provide theoretical guidance for clinicians to revascularize the femoral head in femoral neck fractures.

## Conclusion

A vascular corrosion cast of the femoral head can be made by microperfusion of the retinacular arteries with epoxy resins followed by corrosion of the bone with acid and alkali. The origin, course and branches, and diameter, as well as the anastomosis of nutrient arteries in femoral head, can be clearly shown. This study provides an experimental technique for further investigating the vascular network in the human femoral head and for exploring methods to improve the blood supply of the femoral head after femoral neck fracture.

## Data Availability

All the data and materials are available upon request from the corresponding author.

## Authors' contributions

All authors had full access to the data in the study and take responsibility for the integrity of the data and the accuracy of the data analysis. Conceptualization: RuiXing Hou and JiHui Ju. Methodology: XiangNan Zhang and Wei Deng. Investigation: XiangNan Zhang, Songqiang Zhang, and HongYu Wang. Formal Analysis: XiangNan Zhang and DingSong Wang. Resources: XiangNan Zhang and KaiLong Geng. Writing—Original Draft: XiangNan Zhang and Wei Deng. Writing—Review and Editing: XiangNan Zhang, GuangLiang Zhang, and YingYing Le. Visualization: JiHui Ju. Supervision: JiHui Ju. Funding Acquisition: RuiXing Hou.

## Compliance with Ethical Standards

## Conflict of Interest

All the authors declare they have no conflicts of interest.

## Ethics Approval

This study was approved by the Ethics Committee of Ruihua Hospital affiliated with Soochow University, approval number RX2019003.

## Supporting information


**Video 1**. Three‐dimensional distribution video of internal arteries in swine femoral head.
